# A Case of Eosinophilic Gastroenteritis with Ascites

**DOI:** 10.1155/2015/971607

**Published:** 2015-12-24

**Authors:** Erkan Caglar, Aslı Sezgin Caglar, Suut Gokturk, Ahmet Dobrucali

**Affiliations:** ^1^Cerrahpasa Medical Faculty, Gastroenterology Department, Istanbul University, 34098 Istanbul, Turkey; ^2^Endocrinology Department, Erciyes University, 38280 Kayseri, Turkey; ^3^Bakirkoy Training and Education Hospital, Clinic of Gastroenterology, 34147 Istanbul, Turkey

## Abstract

Eosinophilic gastroenteritis is a rare disorder of unknown cause characterized by focal or diffuse eosinophilic infiltration of gastrointestinal tract, especially the stomach and duodenum. Its clinical presentation depends on which segment of gastrointestinal tract is affected and on the depth of eosinophilic infiltration in the affected segment. We present a case of a 35-year-old male with abdominal distention for six months. Laboratory testing revealed elevated eosinophil count and serum immunoglobulin E (IgE) levels. In abdominal tomography, ascites was observed, and eosinophilic infiltration was detected in duodenum biopsy samples, collected during endoscopic examination of upper gastrointestinal system. Clinical and pathologic findings of the patient responded to steroid dramatically. Even though their comorbidity is rare, eosinophilic gastroenteritis should be considered in differential diagnosis of patients with unspecified ascites.

## 1. Introduction

Eosinophilic gastroenteritis (EG) is a rare disorder of unknown etiology that affects both adults and children. Although cases have been reported worldwide, the exact incidence of eosinophilic gastroenteritis is unclear [1]. It can present with a broad spectrum of symptoms; it may affect essentially any part of the gastrointestinal system and can involve any or all layers of the gastrointestinal wall. Klein et al. have showed that this disorder could be pathologically classified into three major subtypes, affecting predominantly the mucosal layer, muscle layer, or subserosal layer [2]. Patients with serosal type frequently have ascites, which may lead to the wrong diagnosis of EG due to unusual clinical manifestation. Therefore, it is an easily omitted situation that needs more consciousness of the internists and gastroenterologists for diagnosis.

## 2. Case

**Figure 1:**
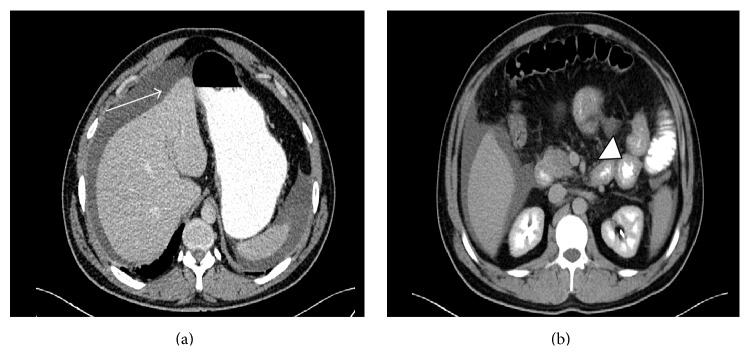
(a) Abdominal CT with oral and intravenous contrast demonstrated ascites in perisplenic and perihepatic area (white arrow). (b) Image study shows multiple segment of small bowel wall thickening (white arrow head).

**Figure 2:**
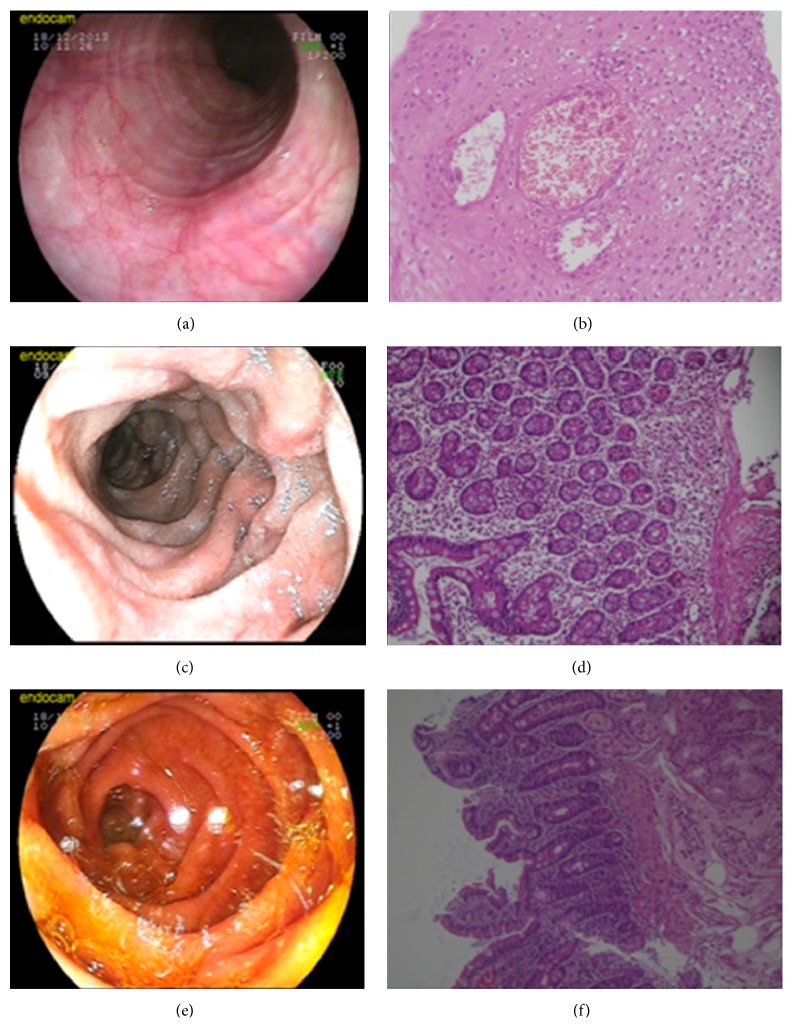
(a) Endoscopy reveals concentric rings (felinization). (b) Mucosal biopsy showed eosinophilic microabscess in the squamous epithelium (H&E stain, ×200). (c) Endoscopic examination showed extensive congestion and edema in duodenum. (d) Mucosal biopsy demonstrated eosinophilic infiltration with inflammatory cells in the lamina propria and submucosa (H&E stain, ×200). (e) On the follow-up endoscopy after 3 months, the duodenal mucosal congestion and edema are improved. (f) Biopsies were obtained at follow-up endoscopy that shows normal mucosal architecture (H&E stain, ×200).

A 35-year-old male presented with acute onset nausea and abdominal distention. He had a history of bronchial asthma and allergic rhinitis; however, he had no history of any other autoimmune disease. He was taking no other medications. Physical examination showed abdominal distension. There was no organomegaly or abdominal mass. Laboratory tests on admission revealed leukocytosis (eosinophil: 6370/mm^3^%), high serum levels of IgE, and eosinophilic cationic protein (ECP). Serum electrolytes, coagulation studies, thyroid, kidney, and liver tests were normal. Stool examinations were negative for parasites, ova, and other common pathogens. Computerized tomography of chest was normal. Abdominopelvic computerized tomography with oral-IV contrast showed moderate ascites with the thickening of small bowel wall (Figure 1). Further, he underwent esophagogastroduodenoscopy (EGD) which showed felinization of the esophagus and extensive congestion and edema in the duodenum (Figures 2(a) and 2(c)). Biopsies demonstrated significant eosinophilic infiltration in the duodenum and esophagus (hematoxylin and eosin stain, ×200, Figures 2(b) and 2(d)). Colonoscopy revealed no abnormality. His blood tests for determining specific IgE levels for food sensitivity were positive to seafood, carrot, and yeast. Bone marrow biopsy showed no abnormalities. The patient was treated with 40 mg prednisolone and seafood, carrot, and yeast were excluded from his diet. Prednisolone was tapered over 8 weeks and continued with 5 mg prednisolone daily. After the steroid therapy, the abdominal pain and physical finding of ascites completely disappeared and blood tests revealed decrease of absolute eosinophil count (<120/mm^3^) and normalization of serum IgE and ECP levels to below upper limit of normal (see Table 1). After 3 months, the EGD control demonstrated normal duodenal mucosa (Figures 2(e) and 2(f)). Resolved eosinophilic infiltration was detected in histologic examination.

## 3. Discussion

Eosinophilic gastroenteritis (EG) is a rarely observed disease of unknown origin. The diagnosis of EG is difficult due to its various form of clinical presentation, and it requires a high index of clinical suspicion. EG should be included in differential diagnosis in patients with peripheral eosinophilia, applied with gastrointestinal symptoms, because eosinophilia is observed at peripheral blood in 30% to 80% of the cases [3]. History of atopy is present in 80% of the patients with eosinophilic gastroenteritis [4]. In the literature, histories of atopic diseases such as asthma, eosinophilic dermatitis, food intolerance, and drug allergy have been reported in cases of eosinophilic gastroenteritis presenting with ascites [5–7]. The patient, our patient, had peripheral blood eosinophilia at presentation and had also a history of allergic rhinitis and asthma.

Most patients with EG have evidence of positive skin allergen test to a variety of food allergens without any anaphylactic reaction. The immune response to various food allergens in the settings of EG is considered to be a delayed type of hypersensitivity reaction, the so-called “Th2 immune response” [8]. Although a definite triggering cause is not known (toxins and bacterial and viral antigens, etc.), food allergens may stimulate the transformation of lymphocytes in the gastrointestinal lymphoid tissue to cytokine secreting Th2 effector cells or IgE producing plasma cells in sensitive persons [9]. The most relevant TH2 cytokines are IL-4, IL-5, IL-9, and IL-13. IL-5 is a key mediator in eosinophil activation and is required for the expansion of eosinophils and their migration from bone marrow. Eosinophils are the hallmark of pathology of EG. Eosinophils produce and release highly bioactive inflammatory mediators like eosinophilic cationic proteins (ECP), eosinophilic peroxidase, eosinophil-derived neurotoxin, and major basic protein (MBP). Many studies reported evidence of extracellular accumulation of MBP and ECP in the small intestinal mucosa of patients with EG [3]. The clinical signs and the organ disorders can be attributed to a great degree to such products.

Three types of EG are defined according to Klein classification: mucosal, muscular, and subserosal types. Clinical findings vary depending on the layer involved. Three layers of gastrointestinal tract may be affected concomitantly. Thus, some authors reported that the combination of these types depends on the long-term disease duration [10]. Subserosal type may present with ascites. Our case was admitted with abdominal swelling and nausea, and ascites was detected in physical examination and abdominal ultrasonography. When ascites is sampled in patients with eosinophilic gastroenteritis, a fluid content of exudative form with considerably high eosinophil count is usually present [5, 11]. It is reported that steroid treatment of this ascites is usually dramatic [12]. In our patient, ascites has not been sampled, but his response to corticosteroid therapy supports the diagnosis.

EG produces much less marked macroscopic changes and as such histopathological diagnosis is essential. Endoscopic appearance is not formed of specific lesions, but it rather demonstrates nonspecific findings such as thickened mucosal folds, erythema, edema, erosion, and ulcer [11]. It is diagnosed by endoscopic biopsies or open tissue sampling. Still there is the chance of a negative result, and this should lead to suspected involvement of patch-form. Multiple samplings should be performed in endoscopic biopsies in order not to miss the diagnosis. Leal et al. reported that macroscopic appearance of upper gastrointestinal endoscopic examination of a case presenting was normal, and eosinophil infiltration was not observed in endoscopic biopsies, and that they performed open peritoneal biopsy for diagnosis [5]. In our case, congestion and edema at duodenal mucosa were observed at endoscopy and eosinophilic infiltration at biopsies was detected. The presence of thickened small bowel wall and ascites in abdominopelvic CT suggests that mucosal infiltration is accompanied with serosal involvement in our patient.

When EG is detected in patients with eosinophilia, systemic reasons should be investigated and eliminated. Connective tissue disorders such as systemic lupus erythematosus or scleroderma may cause EG concomitantly present with eosinophilic ascites [13]. Moreover, vasculitis (Churg-Strauss syndrome), malignancy (eosinophilic leukemia, lymphoma), and systemic parasitosis lead to these systemic reasons [11, 14]. There was no clinical and laboratory evidence suggesting a vasculitic syndrome or connective tissue disorder in our patient. Examination of bone marrow biopsy showed normal cellularity. There was no parasite or ova in parasitologic stool examination. A parasitic infection or infestation should completely be eliminated in any EG case prior to the corticosteroid therapy, because steroid treatment in the presence of occult parasitic infection may result in catastrophic disseminated disease.

Although there are some case reports showing that EG may resolve spontaneously without treatment, most of the patients need medical therapy. Treatment includes elimination or elemental diets and drug therapy using classical antiallergic agents. Avoiding the dietary intake of food implicated by skin prick or food allergy test and the use of an elemental diet have been reported to have a variable effect; elimination of presumed dietary articles is unhelpful in most cases [15–18]. In our patient, specific IgE types, formed against food, were investigated in serum, and specific IgE was detected against carrot, seafood, and yeast. Ishimura et al. reported that although the diagnostic value of specific IgE antibody levels is low it is an easy and less invasive method for target antigen detection [19]. Corticosteroid treatment is the major treatment form, and patients typically respond to steroid therapy quite rapidly. It has been reported that patients with early onset of eosinophilic gastroenteritis usually respond dramatically to low dose steroid, and therefore ascites or gastric outlet obstruction, requiring surgery, which emerge at late stage, were prevented [20]. However, relapse may occur in some patients after the discontinuation of steroid treatment. Different therapeutic agents, such as azathioprine, montelukast, ketotifen, and cromoglycate, may offer an alternative therapeutic option in order to avoid the side effects of long-term steroid therapy [11, 21–23]. In recent years, it is reported that monoclonal antibodies developed against IgE and IL-5 are starting to be used successfully in the treatment [24, 25]. Thus, both biochemical improvement and improvement at endoscopies and biopsies were observed in our patient following prednisolone treatment. Long-term prednisolone treatment at low maintenance dose was continued to be administered to the patient. If elevated eosinophil count, serum IgE, and ECP levels have been detected at diagnosis, these parameters should be monitored carefully during the follow-up of the treatment.

In conclusion, EG should be considered in differential diagnosis in patients with ascites accompanied with a history of atopy and blood eosinophilia. Endoscopic examination of gastrointestinal tract should be performed, and, if required, open serosal biopsies should be certainly collected for diagnosis. Corticosteroid treatment is the major therapeutic option, and physician should keep in mind that the disease may have a relapsing course.

## Figures and Tables

**Table 1:** Laboratory results of the patient before and after steroid therapy.

	Before therapy	3 months after therapy
White blood cell (normal: 4000–10000/mm^3^)	17900	9600

Eosinophils (%) (normal: 0–400/mm^3^)	6370 (35.6)	120 (1.3)

Total immunoglobulin E (normal: 0–100 IU/L)	1440	76

Eosinophilic cationic protein (normal: 0–20 ng/mL)	200	51.7
